# Transdifferentiation from cornea to lens in *Xenopus laevis *depends on BMP signalling and involves upregulation of Wnt signalling

**DOI:** 10.1186/1471-213X-11-54

**Published:** 2011-09-06

**Authors:** Robert C Day, Caroline W Beck

**Affiliations:** 1 Genetics Otago, University of Otago, PO Box 56, Dunedin 9054, New Zealand; 2 Department of Biochemistry, University of Otago, PO Box 56, Dunedin 9054, New Zealand; 3 Department of Zoology, University of Otago, PO Box 56, Dunedin 9054, New Zealand

## Abstract

**Background:**

Surgical removal of the lens from larval *Xenopus laevis *results in a rapid transdifferention of central corneal cells to form a new lens. The trigger for this process is understood to be an induction event arising from the unprecedented exposure of the cornea to the vitreous humour that occurs following lens removal. The molecular identity of this trigger is unknown.

**Results:**

Here, we have used a functional transgenic approach to show that BMP signalling is required for lens regeneration and a microarray approach to identify genes that are upregulated specifically during this process. Analysis of the array data strongly implicates Wnt signalling and the Pitx family of transcription factors in the process of cornea to lens transdifferentiation. Our analysis also captured several genes associated with congenital cataract in humans. Pluripotency genes, in contrast, were not upregulated, supporting the idea that corneal cells transdifferentiate without returning to a stem cell state. Several genes from the array were expressed in the forming lens during embryogenesis. One of these, *Nipsnap1*, is a known direct target of BMP signalling.

**Conclusions:**

Our results strongly implicate the developmental Wnt and BMP signalling pathways in the process of cornea to lens transdifferentiation (CLT) in *Xenopus*, and suggest direct transdifferentiation between these two anterior eye tissues.

## Background

Urodele amphibians, for example the axolotl, are well known for their incredible ability to regenerate appendages, such as the limb. However, axolotls are unable to regenerate the lens of the eye following its removal (lentectomy). In contrast, the anuran amphibian *Xenopus laevis*, in which limb regeneration is subject to an ontogenic decline leading up to metamorphosis, is able to regenerate a new lens from the overlying central corneal cells (for review see [1,2]). This process was first described by Freeman in 1963, and involves a transdifferentiation of one cell type (corneal epithelium) to another (lens) [3]. It differs from the better-known Wolffian regeneration in adult newts, where a new lens is formed from cells of the pigmented dorsal iris epithelium and is known as cornea to lens transdifferentiation, or CLT [2].

The trigger for CLT *in vivo *is exposure of the outer corneal cells to an unidentified factor present in the vitreous of the eye, most likely originating from the neural retina [4,5]. *In vitro*, epithelial cells from any location within the lentogenic area, a region extending twice the diameter of the eye [3] can respond to the vitreous factor and initiate CLT, whereas cells outside this region are refractory to the trigger [6-8]. The limitation of lens forming ability to the lentogenic area correlates with *Pax6 *expression, and ectopic *Pax6 *in flank epidermis can confer competence to undergo CLT [9]. As with other cases of regeneration in *Xenopus*, there is an ontogenic decline in the ability to initiate CLT *in vivo *[3], however, this is thought to arise due to a mechanical barrier formed by the healing of the inner cornea rather than a loss of competence [10]. Interestingly, the close relative *Xenopus tropicalis*, which exhibits more rapid healing of the inner cornea following lentectomy, fails to initiate CLT *in vivo *although reciprocal transplants show that the central corneal cells of *X. tropicalis *can respond to the vitreous factor [11].

Fifty years on from the discovery of CLT, we still know little of the molecular mechanisms that drive the process. While it is generally believed that transdifferentiation occurs directly and not via proliferation of stem cells [12], a direct demonstration of this is lacking. Jon Henry and colleagues have shown that several transcription factors known to be fundamental to lens development are re-expressed during the process of CLT (Pax6, Prox1, Otx2 and Sox3), suggesting that similar regulation of gene expression drives differentiation during both development and regeneration of the lens [13,14]. Previous EST analysis of corneal cells undergoing CLT has identified several hundred transcripts from a library constructed from corneal tissue at 1-4 days after lens removal [14,15].

Despite the identification of multiple candidate pathways from these expression studies, functional analysis of potential transdifferentiation factors has so far been lacking. A single *in vitro *study demonstrated the ability of acidic fibroblast growth factor (aFGF) to induce lens fibre formation in cultured outer corneas, although morphological organisation of the fibres does not occur [16]. In the current study, we have used a transgenic line of *Xenopus laevis *to reveal a need for functional BMP signalling during the process of CLT along with a microarray strategy to identify genes and pathways that are likely to be specifically involved in the process of transdifferentiation. The microarray strategy differs from previous library based approaches in that we can specifically compare expression in wounded, non-regenerating corneas to that in corneal tissue undergoing CLT, with the aim of identifying genes associated with the regenerative process. Analysis of this microarray data indicates an important role for wingless/int1 (Wnt) pathway signalling in CLT and suggests that, as with tail and limb regeneration in *Xenopus *[17-19], several morphogens may be acting to trigger the regenerative process of CLT. We have identified several new candidates for CLT, some of which are also involved in lens formation during development, and many of which are associated with lens pathology, particularly cataract development.

## Results

### Lens regeneration is dependent on BMP signalling

**Figure 1:**
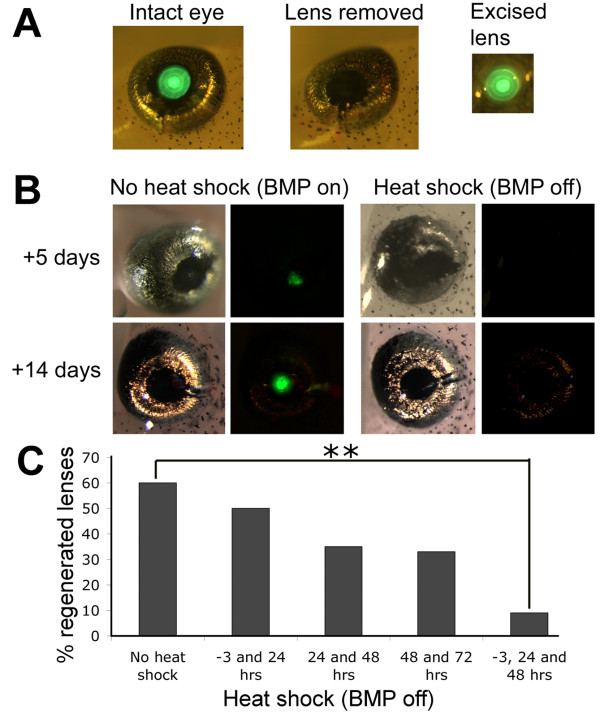
**BMP signalling is required for lens regeneration**. A) Lentectomy of N1 transgenic tadpoles at stage 50. These tadpoles carry the *Hsp70:Noggin; γ-Crystallin:GFP *transgene and express GFP in the central lens cells, but not the outermost lens epithelial cells. Removal of the lens can be visualised by the lack of GFP in the lentectomised eye. B) Heat shock initiates expression of the BMP inhibitor noggin, attenuating BMP signalling. In around 60-70% of non heat shocked, lentectomised eyes, regeneration of the lens from the overlying cornea occurs and GFP can be seen after 3-5 days and a new lens is formed after 14 days. When the tadpoles are subjected to heat shock, no GFP is expressed and no lens regenerated. Bright field views are to the left with corresponding fluorescent views adjacent. C) Graph showing the effect of heat shock activation of Noggin on lens regeneration as determined by the presence of any detectable GFP in the eye 10 days after lentectomy. ** indicates p < 0.001. (Chi squared analysis).

We have previously shown that overexpression of *Noggin*, an inhibitor of BMP signalling, can prevent regeneration of tails and limbs in *Xenopus *tadpoles [17,20]. The stable transgenic line, N1 contains a single insertion of the double transgene *Hsp70:Noggin;γ-Crystallin:GFP*. The eyes of N1 tadpoles are marked by green fluorescent protein (GFP), which accumulates in the inner lens cells regardless of the temperature at which the animals are kept (Figure 1A). When the GFP expressing lens is surgically removed and the cornea replaced, no green fluorescence can be detected. If lentectomised N1 tadpoles are raised at 24°C, GFP is first detected after 3-5 days, indicating that CLT is underway and new lens cells are expressing *γ-Crystallin *(Figure 1B). In our hands, CLT occurs in 60-70% of cases, and after two weeks, the new lens is morphologically indistinguishable from the original. However, if the N1 tadpoles are subjected to heat shock, by placing in water at 34°C for 30 minutes each day, CLT usually fails, and no new expression of GFP is detected, suggesting that BMP signalling must be active for transdifferentiation to occur (Figure 1B). The process of heat shocking itself did not adversely affect the regenerative potential of wild type sibling eyes or those carrying a γ-cry-RFP transgene (C.B. unpublished observations).

**Figure 2:**
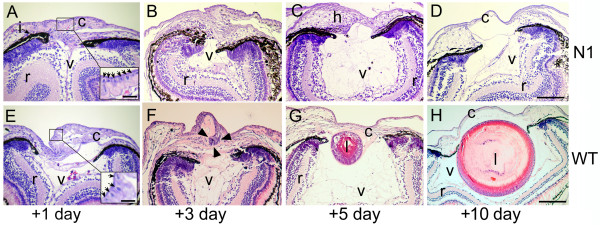
**Histology of WT and BMP inhibited (N1) tadpole eyes following lentectomy**. (A-D) Representative histological sections through the eyes of transgenic N1 tadpoles 1, 3, 5 and 7 days after lentectomy. Inset in A shows arrows pointing to columnar corneal cells (E-H) Representative sections of wild type eyes at 1, 3, 5 and 7 days after lentectomy. Inset in E indicates columnar corneal cells (arrows). Arrowheads in F indicate the forming lentoid moving into the vitreous. Both transgenic and wild type eyes were subjected to heat shock at -3, 24 and 48 hours relative to lentectomy. Abbreviations: c, cornea; h, hypertrophic cells; i, iris (pigmented epithelium); l, lens; r, retina; v, vitreous. Scale bars in D and H corresponds to 50 μm and applies to all panels. Inset scale bar in A and E corresponds to 10 μm.

We have previously shown that each daily heat shock generates a burst of *Noggin *expression that is detectable for a day or two, but declines[20]. Heat shocks were applied at different times during post lentectomy recovery (Figure 1C). Heat shock at -3, +24 and +48 hours, relative to surgical removal of the lens, resulted in only 9% of eyes regenerating detectable GFP positive lens cells (n = 56), compared to non heat shocked transgenics of which 60% regenerated lens cells (n = 60). This difference was found to be highly significant by chi squared analysis (p < 0.001). Tadpoles heat shocked twice, at either 24 and 48 hours or 48 and 72 hours, regenerated lens in 35% (n = 23) and 33% (n = 15) of cases respectively, and chi squared analysis compared to unheatshocked controls showed that this was close to significant (p = 0.06 and 0.09 respectively). Heat shock of tadpoles at -3 and 24 hours resulted in 50% regeneration (n = 10), suggesting that BMP signalling needs to be sustained to inhibit lens regeneration fully. To investigate this further, we sectioned lentectomised eyes one, three, five and ten days after lentectomy and compared the histology of heat shocked N1 tadpoles to that or similarly treated WT sibling animals. Heat shocks were applied before lentectomy and for the first two days afterwards (unless fixed at day 1) (Figure 2). Freeman described five distinct phases of CLT in *Xenopus laevis *based on histological analysis [3]. At stage 1 (1-2 days after lentectomy), cells of the inner layer of the outer cornea have undergone a change from squamous to cuboidal epithelium. In both N1 and WT eyes one day after lentectomy, corresponding to stage 1, the vitreous appeared collapsed and the cornea very thickened (compare Figure 2A and 2E to 2H), with no obvious differences between the transgenic and WT eyes. Cuboidal cells were consistently visible in both cases (Figure 2A and 2E insets), suggesting that this initial stage of CLT does not depend on BMP signalling. Indeed, Freeman observed that this stage occurs on wounding of the cornea even if the lens is not subsequently removed [3]. We observed a slightly thicker eosin stained extracellular matrix in some, but not all, N1 samples. In later stages, though, a clear difference in the histology of the WT and N1 eyes was observed. In N1 tadpoles, in which BMP signalling is attenuated, the cells of the thickened cornea appear to become hypertrophic (Figure 2B, C). By ten days the cornea has thinned to its original state, with the cells once again appearing squamous, and the vitreous has partially reinflated despite the lack of a lens (Figure 2D). Of 8 ten day samples sectioned, one showed regeneration of a small lentoid.

In WT eyes fixed three days post lentectomy, in 5/10 samples, the transdifferenting cornea had reached early Freeman stage 3, and a cluster of aggregated cells is beginning to invade the vitreous (Figure 2F). A further 2/10 had reached mid to late Freeman stage 3 and no CLT was observed in the remaining 3 samples. At this stage the connection to the cornea is very clear. By five days, the aggregate has detached from the cornea and become a lens vesicle and primary lens fibres have begun to form (Figure 2G, Freeman stage 4). By ten days, primary and secondary lens fibres have formed and the lens appears essentially complete (Figure 2H, Freeman stage 5). The cornea has returned to its original state and is once more composed of squamous epithelial cells.

### Microarray analysis of CLT I: Pattern matching based on crystallin expression (CRY list)

**Figure 3:**
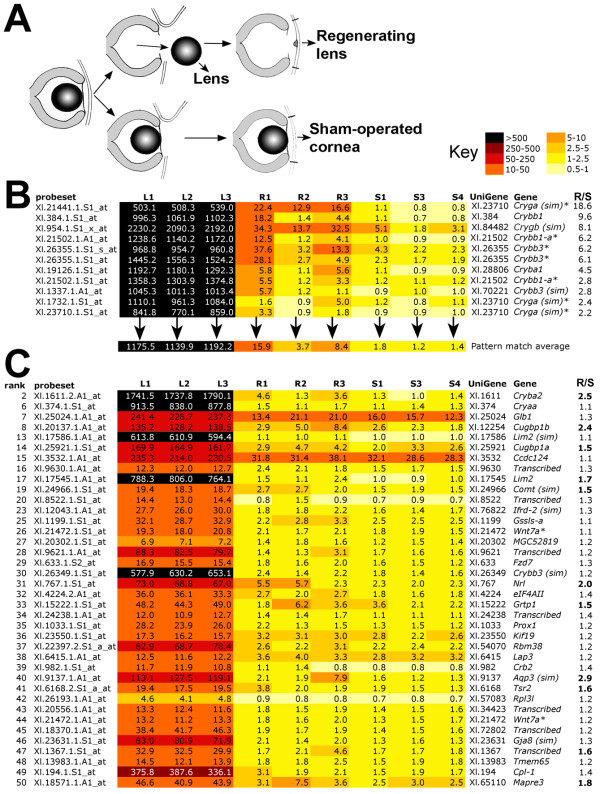
**Identification of genes that mimic expression of crystallins during CLT**. A) Strategy for identifying CLT specific genes by microarray. Lenses were removed from stage 50 tadpoles and used for RNA extraction of samples L1-3 (differentiated lens). The tadpoles that had undergone lentectomy were allowed to recover for three days before harvesting the cornea for samples R1-3 (regenerating). Finally, a second set of tadpoles were subjected to mock surgery, the cornea was cut, lifted and replaced as for lentectomy but the inner cornea and lens were left in situ. After 3 days corneas were recovered from these animals, generating samples S1, 3 and 4 (sham operated). B) Heatmap of Affymetrix array data showing that the pattern of crystallin expression varies among replicate R samples, with R2 consistently lower. An average expression was used to search for similar patterns in the data. C) Heatmap of top 50 significant matches to the crystallin pattern, excluding the crystallin genes used to generate the pattern average. R/S is the ratio of transcript expression in R1-3 vs. S1, 3 and 4 levels. Bold type in C indicates R > 1.5 × S.

Microarray samples were prepared as shown in Figure 3A. Nine Affymetrix *Xenopus laevis *GeneChips were probed with triplicate biological replicates prepared either from stage 50 lens (L), corneas undergoing CLT (expected to be predominantly at early Freeman stage 3) (R) and sham operated corneas undergoing healing (S). The normalised data were first sorted by performing pattern matching using TIGR-MEV with the instruction that R (mean) is greater than any of the three S replicates. Thus, the average intensity for a particular probeset from three biological replicate samples of CLT tissue should be statistically higher than for any of the sham operated controls, in which wound healing but not CLT is occurring. At the top of this list were several probesets representing members of the crystallin (Cry) family of structural lens proteins. However, between biological replicates, there was a pattern among the crystallins in the CLT replicates whereby R1 and R3 were consistently seen to have higher expression than R2. We took this to be an indication that, in the pool of corneas that made up the R2 sample, CLT was either lagging behind or occurring in fewer of the corneas. We made an assumption that other genes being specifically switched on during CLT might follow the same pattern, and so we selected 11 probesets representing seven different UniGene sets of crystallins, and averaged the intensities for each sample (Figure 3B). These average intensities were then used with TIGR-MEV pattern matching software to search for similar patterns across the 9 samples. 1642 statistically significant (p < 0.05) matches were obtained (CRY list). The top 50 ranked matches were manually annotated and are shown in the heat map in Figure 3C. The full CRY list can be found in additional file 1 (note this is not annotated fully).

Crystallins used in the pattern match are shown in Figure 3B. Three further crystallin genes, *Cryba2, Cryaa *and a gene most similar to *Crybb3*, were detected by the match. *Prox1*, a known eye development gene, was also captured. The Wnt family member Wnt7a appeared twice (due to the presence of multiple probe sets captured by the filter) in the top 50, along with the Wnt receptor Frz7, which occurred a total of four times in the CRY list. Seven of the top 50 ranked genes could not be assigned to a protein family and are annotated as *transcribed*. Interestingly, two homologues of a CUG triplet repeat RNA binding protein, Cugbp1, were also ranked highly in the match. These proteins, also known as EDEN-BP, are members of the Bruno family and are involved in degradation of mRNA through binding to instability sites [21].

To obtain an unbiased overall view of the selected crystallin pattern matched data, 1037 genes with a high probability match (p < 0.01) were used to examine gene ontology. These formed the slightly shorter CRY* list. TC numbers, with duplicates removed, were used to match probesets to specific GO biological function categories. Table 1 shows the GO categories that were statistically over represented in the CRY* dataset. Genes associated with nuclear migration, microtubule polymerisation, development, Wnt/planar cell polarity (PCP) signalling, eye development and visual perception were among the most over represented ontologies. The two genes in "eye development (*sensu vertebrata*)" correspond to the lens intrinsic membrane protein *Lim2 *and a possible homologue, *Lim2(sim)*. Lim2 encodes a lens specific protein probably involved in cell junctions, and mutations in humans are associated with congenital cataracts [22,23]. The inclusion of two categories involved with Wnt/PCP signalling, "R3/R4 cell fate commitment" and "establishment of wing hair orientation" ontologies seems to be entirely due to one gene, *Frz7*, which has two TC numbers, and a total of 4 hits in the list. However, the "frizzled signalling pathway" ontology was also significantly overrepresented (Table 1), creating a strong case for the involvement of Wnt signalling in directing the lens fate. Genes in this category included, as well as *Frz7*, a second receptor gene, *Frz8 *(2 hits), the ligand *Wnt5b *transcription factors *Pitx3 *and *Tcf3*, and the signalling components *Axin1 *and the alpha subunit of *CK2*.

**Table 1:** Top 10 statistically over represented GO categories in the CRY* list

GMRG_Term	TC numbers		p	Description
			
	total	subset		
GO:0000065/0000743	16	6	0.001	nuclear migration along microtubule/during conjugation with cellular fusion
GO:0007275	243	32	0.003	multicellular organismal development
GO:0046785	10	4	0.006	microtubule polymerization
GO:0001737	2	2	0.006	establishment of wing hair orientation
GO:0006221	2	2	0.006	pyrimidine nucleotide biosynthetic process
GO:0007464	2	2	0.006	R3/R4 cell fate commitment
GO:0043010	2	2	0.006	eye development (sensu Vertebrata)
GO:0006364	29	7	0.006	rRNA processing
GO:0007601	87	14	0.008	visual perception
GO:0007222	12	4	0.012	frizzled signaling pathway

TC numbers are tentative consensus numbers and each one represents a group of ESTs for one gene, as well as providing a way to link to gene ontology. Total TC therefore shows the number of genes on the *Xenopus *GeneChip which are linked to a particular ontology, and subset TC shows how many of these were found in the CRY* list

Several genes were validated using quantitative real time PCR (q-rtPCR), as shown in Table 2. After normalisation to the housekeeping gene ODC, 11/11 genes were confirmed as being expressed at higher levels in lens than in either corneas undergoing CLT or sham operated corneas. Of these, six were confirmed expressed at higher levels in corneas undergoing CLT vs. sham operated corneas: five crystallins and *Fzd7*. Two of the remaining genes (*Wnt7a, Kif19*) were found to be downregulated by q-rtPCR and unchanged in the array, *Glb1 *was unchanged in either analysis, and *Cryaa *was found to be upregulated by q-rtPCR but not on the array. The RNA binding protein Cugbp1b was not significantly up regulated by q-rtPCR although expression was confirmed as being higher in CLT samples.

**Table 2:** Q-rtPCR validation of 11 genes from the CRY list

Rank in array	Gene name	Lens (L/S)	CLT (R/S)	
		
		qPCR	qPCR	array
1	*Cryba1*	**37009.6**	**38.5**	**4.5**
3	*Crygb (sim)*	**75864.3**	**7.3**	**8.1**
4	*Crybb3*	**45918.7**	**7.8**	**6.2**
6	*Cryaa*	**95666.6**	**17.9**	1.1
7	*Glb1*	**7.6**	0.8	1.3
8	*Cugbp1b*	**41.8**	1.3	**2.4**
15	*Ccdc124*	**3.62**	**1.5**	1.1
18	*Crybb1*	**22333.3**	**5.0**	**9.6**
26	*Wnt7a*	**39193.7**	*0.5*	1.1
29	Fzd7*	**5.9**	**3.9**	1.3/**2.7**
36	*Kif19*	**2.1**	*0.5*	1.2

Bold numbers indicate upregulation of more than 1.5 fold, numbers in italics indicate down regulation and normal font indicates no significant change. * appears twice in CRY list. L/S shows the relative (fold higher) expression in lens when compared to sham operated corneas. Likewise R/S shows relative expression in corneas undergoing CLT when compared to sham operated control corneas.

**Figure 4:**
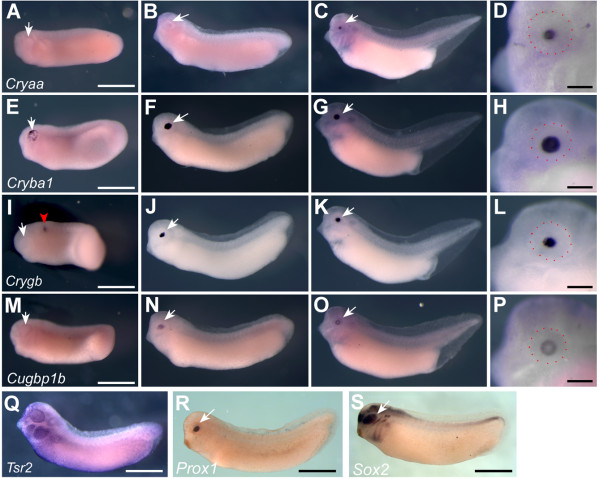
**Expression of lens specific genes during development**. *In situ *hybridisation of three *Crystallin *genes and *Cugbp1b *during development of the lens, indicated by dark blue staining. A-D, *Cryaa *expression at st. 26 (A), 30 (B) and 38 (C and D). E-H, Expression of *Cryba1 *throughout the lens at st. 26 (E), 30 (F) and 38 (G and H). I-L, expression of *Crygb*. I) at st. 26, note that red arrowhead indicates a transient stripe of expression in the hindbrain. *Crygb *is expressed in the central lens at st. 30 (J) and 38 (K and L). M-P) *Cugbp1b *transcripts are absent from the forming eye at st 26 (M), detected in the central lens cells at st.30 (N), and in the outer lens cells at st. 38 (O and P). Q) *Tsr2 *is expressed in epithelial cells including the cells overlying the eye, shown at stage 30. R) *Prox1 *expression is lens specific at stage 30, as previously described [23]. *Sox2 *is expressed throughout the eye as well as in the olfactory placode lateral line primordia and branchial arches as previously described [24]. White arrows indicate the position of the eye, scale bars in D, H, L, P, Q, R and S are 200 μm and red dots show the approximate margin of the eye. Scale bars in A, E, I and M apply to all other panels and correspond to 1 mm. Embryos are oriented with anterior to the left and dorsal uppermost.

Seven genes from the CRY list were then cloned and used to determine developmental expression patterns using *in situ *hybridisation (Figure 4). *Cryaa *was expressed in central cells of the developing lens from stage 30 (Figure 4A-D) corresponding to the primary lens fibres. *Cryba1 *was expressed much earlier in lens development, and was first seen in punctate pattern across the whole lens placode at stage 26 (Figure 4E). Expression appeared to be in all lens cells at stage 30 (Figure 4F) and this was maintained at stage 38 (Figure 4G, H). *Crygb *was first detected in the hindbrain in a single stripe at stage 26 (Figure 4I). This expression was transient and was superceded by expression in the central two thirds of the lens cells by stage 30 (Figure 4J). This expression was maintained at stage 38 (Figure 4K, L). *Cugbp1b *expression was first seen at stage 30, specifically in lens cells (Figure 4M, N). By stage 38, expression had cleared from the central, *Cryaa *expressing cells, but was maintained in peripheral lens cells corresponding to the secondary lens fibres (Figure 4O, P). *Tsr2 *transcripts were detected in epithelial cells and were not specific to the eye (Figure 4Q). Two previously described eye genes also found in the CRY list, *Prox1 *and *Sox2*, were included here for comparison. *Prox1 *transcripts were seen specifically in lens cells at stage 30 as previously described [24]. *Sox2 *(Figure 4R) expression was seen in optic cup and lens as well as in the forming lateral line, branchial arches and neural tube (Figure 4S) [25].

### Microarray analysis of CLT II: Identification of regeneration associated genes (RAG)

**Figure 5:**
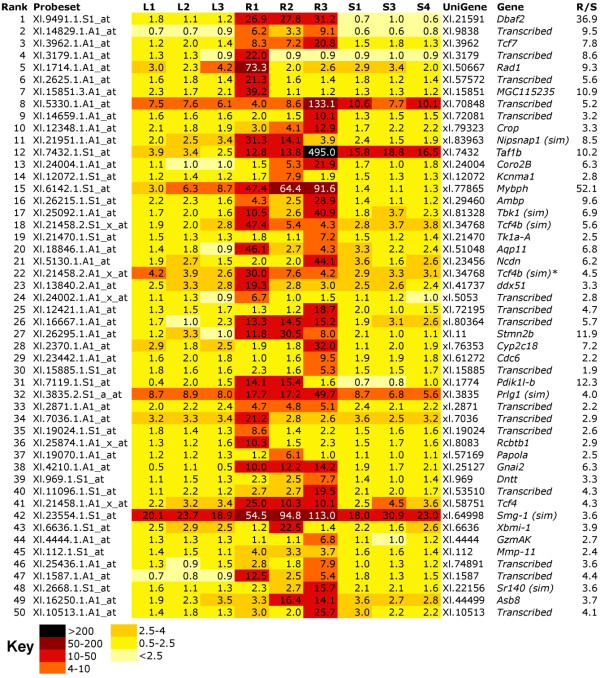
**Heat map of top 50 genes, where expression is higher in samples undergoing CLT (R) than in stage matched sham operated corneas (S) or lenses (L)**. Darker colour indicates higher expression. R/S indicates the ratio of transcript expression in R1-3 vs. S1, 3 and 4 levels. Where possible, the genes have been manually annotated with reference to the probe source files. "Transcribed" is used to denote genes where no annotation was possible. "(sim)" indicates high similarity to the named gene.

A second pattern match was devised to capture potential regeneration associated genes that were not highly expressed in lens (RAG list). The parameters for this were set as low expression in lens, high in samples undergoing CLT (R) and low in sham operated controls (S). 1516 probesets passed this filter with a cut-off *p *value of < 0.05 and the complete RAG list can be found in additional file 2. The top 50 ranked genes were re-annotated manually and their expression profile is shown in Figure 5. Sixteen of the 50 genes could not be annotated and are listed as *transcribed*. The top ranking gene *DBAf2 *encodes the *Xenopus *MHC class II α chain, which could suggest a sustained immune response in CLT. Of particular interest is the third ranking gene, *Tcf7*, also known as *Tcf1 *in *Xenopus*. Tcf7 is a HMG-box transcription factor that represses target gene expression in the absence of Wnt signalling [26].

Also included in the RAG list were the matrix metalloproteinases Mmp11 and Mmp14, transcription factors Pitx2, Pitx2a, Six1, Pax6, and the signalling proteins Fgf8b and Wnt6, which both appear twice. *Tcf4*, which encodes a bHLH transcription factor unrelated to Wnt signalling, appears three times on the list and a closely related gene that we have annotated as *Tcf4b (similar) *appears twice.

Gene ontology of 794 unique TC numbers corresponding to high probability hits (p < 0.01) from the RAG list was analysed (RAG*). Statistically over-represented gene ontologies are shown in Table 3. Strikingly, the most significantly over-represented ontology group was the Wnt receptor signalling pathway. Eight from a possible 41 genes assigned to this group were present in the RAG* list. These genes included three members of the Pitx family of transcription factors, *Pitx1, Pitx2 *and *Pitx2a *as well as Wnt pathway components *Dvl2 *and *Lrp6*, each of which is present twice in the RAG list. Also included in this group were the genes encoding the ligand Wnt2, the HMG group transcription factor Tcf7l2 (appears twice, formerly known as Tcf4 but not to be confused with the bHLH transcription factor described above) and Btrc, an F-box/WD-40 protein associated with ubiquitination. The ontology analysis is not exhaustive: Wnt3 and Wnt6 were also present in the RAG list but had not been assigned a TC number, and were therefore not picked up by the ontology screen. As well as Wnt signalling, two other gene ontology terms, "patterning of blood vessels" and "chromatin assembly or disassembly" were also highly significantly over-represented (Table 3). The gene ontology term "patterning of blood vessels" scored highly because of inclusion of the *Pitx *genes *1, 2 *and *2a*, which are also involved in this process. The genes in this category "chromatin assembly or disassembly" that were present in the analysis were *Unkempt1*, *Histone 2 ab *and *2a*, *Smarcc2*, *Mta2*, *Hsp90ab1 *and *Cbx1*.

**Table 3:** Top 10 statistically over represented GO categories in the RAG* list.

GMRG_Term	TC numbers		p	Description
			
	total	subset		
GO:0016055	41	8	0.003	Wnt receptor signaling pathway
GO:0001569	6	3	0.004	patterning of blood vessels
GO:0006333	40	7	0.009	chromatin assembly or disassembly
GO:0030001	3	2	0.011	metal ion transport
GO:0045010	3	2	0.011	actin nucleation
GO:0006729	3	2	0.011	tetrahydrobiopterin biosynthetic process
GO:0009591	15	4	0.011	perception of mechanical stimulus
GO:0007368	10	3	0.019	determination of left/right symmetry
GO:0006826	10	3	0.019	iron ion transport
GO:0050930	4	2	0.020	induction of positive chemotaxis

**Figure 6:**
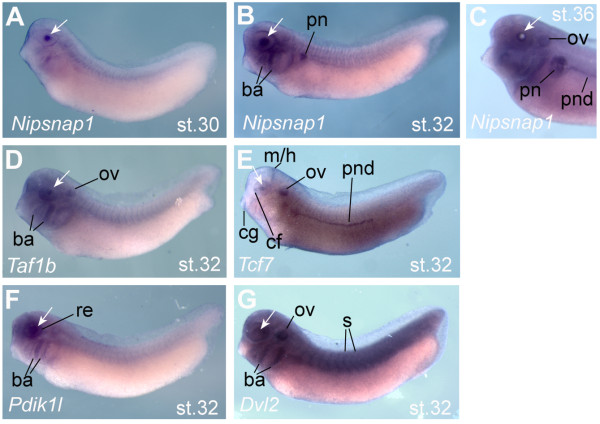
**Expression of selected transdifferentiation genes during development**. In situ hybridisation of five regeneration associated genes (RAG). Dark blue staining marks expression, and a white arrow indicates the position of the lens. A) *Nipsnap1 *expression appears in lens cells at stage 30 (A) and is also found in the pronephros (pn) and branchial arches (ba) from stage 32 (B). At stage 36, *Nipsnap1 *expression is seen in the pronephros and pronephritic duct (pnd) and otic vesicle (ov) as well as in the periphery of the lens (C). D) *Tbp1b *is expressed predominantly in lens, otic vesicle and branchial arches at stage 32. E) *Tcf7 *is expressed in lens and choroid fissure of the retina (cf), the midbrain-hindbrain junction (m/h), cement gland (cg), otic vesicle and pronephritic duct at at stage 32. F) *Pdik1l *is expressed in the developing lens and retina (re) and branchial arches at stage 32. G) *Dvl2 *is expressed in eye tissues but is not specific, with expression extending throughout the embryo including the somites (s), otic vesicle and branchial arches. Embryos are oriented with anterior to the left and dorsal uppermost.

Validation was performed for 13 genes with 9 confirmed as statistically increased in CLT and two more showing the same trend as the array (Table 4). Five genes were examined for expression in developing lens. Although none of these were found to be entirely lens specific at tailbud stages, the BMP target gene *Nipsnap1 *was expressed only in the lens at stage 30, with expression in branchial arches, otic vesicle and pronephros appearing around stage 32 (Figure 6A-C). *Taf1b *was expressed in the lens, branchial arches and otic vesicle from stage 32 (Figure 6D). The Wnt target gene and pathway-specific transcription factor *Tcf7 *was also expressed in developing lens at stage 32 but was also seen in the choroid fissure of the eye cup, midbrain hindbrain boundary, otic vesicle, cement gland and pronephritic duct at stage 32 (Figure 6E). *Pdik1l *expression was restricted to the developing eye (lens and retina) and branchial arches, beginning at stage 30, (Figure 6F) and the Wnt pathway gene *Dvl2 *was expressed almost ubiquitously (Figure 6G).

**Table 4:** Q-rtPCR validation of 13 genes from the RAG list.

Rank in array	Gene name	Lens (L/S)	CLT (R/S)	
		
		qPCR	qPCR	array
3	*Tcf7*	0.6	**5.7**	**7.8**
10	*Crop*	0.7	1.4	**3.3**
11	*Nipsnap1 (sim)*	*0.1*	**176.3**	**8.5**
12	*Taf1b*	1.0	**8.0**	**10.2**
15	*Mybph*	*0.0*	*0.2*	**52.1**
31	*Pdik1l-b*	1.1	**10.3**	**12.3**
38	*Gnai2*	**5.5**	**56.1**	**6.3**
41	*Tcf4*	*0.0*	**4.4**	**4.3**
45	*Mmp-11*	**1.8**	**100.0**	**2.4**
56	*Creld1*	*0.0*	1.6	**5.2**
71	*Amph*	**24.2**	**74.6**	**2.9**
479	*Mmp14*	*0.1*	**3.4**	**2.3**
1391	*Lmo4*	**4.11**	**2.2**	1.4

Bold numbers indicate upregulation of more than 1.5 fold, numbers in italics indicate down regulation and normal font indicates no significant change. L/S shows the relative (fold higher) expression in lens when compared to sham operated corneas. Likewise R/S shows relative expression in corneas undergoing CLT when compared to sham operated control corneas.

### Pluripotency genes are not upregulated in 3 day corneas undergoing CLT

**Figure 7:**
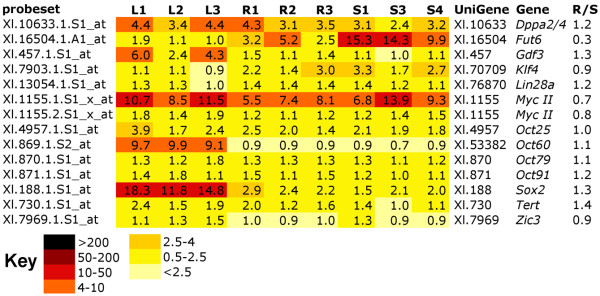
**Heat Map showing that expression of pluripotency associated genes does not change during CLT**. Darker colour indicates higher levels of expression. R/S indicates the ratio of transcript expression in R1-3 vs. S1, 3 and 4 levels. Where possible, the genes have been manually annotated with reference to the probe source files.

Thirteen genes associated with pluripotency were present on the GeneChip array. These included *Klf4 (biklf-A), c-Myc, Sox2*, and four POU genes, *Oct25, Oct60, Oct79 and Oct91*, which seem, in *Xenopus*, to substitute for the pluripotency gene *Oct3/4 *of mammals [27], *Dppa2/4, Gdf3, Fut6 *(*Ssea1 *homologue), *Lin28, Tert and Zic3*. However, with the exception of *Sox2*, none of these were captured in our screen (Figure 7). *Sox2 *was expressed at highest levels in the lens samples (8 fold higher levels than in sham operated cornea controls) but was not significantly elevated in corneas undergoing CLT when compared to the same controls. *Sox2 *then may be involved in lens differentiation but does not seem to be indicative of dedifferentiation in this case. Another pluripotency associated gene, *Fut6*, was significantly upregulated in sham operated corneas when compared to corneas undergoing CLT (3.7 fold increase), with no expression in lens. Since we only observed one time point, it is possible that we have missed a change in pluripotency gene expression. Further analysis of earlier and later stages of CLT will be needed to exclude this possibility.

## Discussion

### BMP signalling is essential for CLT

BMP signalling has not been previously linked to transdifferentiation of the cornea into lens. However, Faber et al demonstrated that BMP signalling is required for mammalian primary lens cell differentiation both in vivo and in vitro [28]. Furthermore, BMP4 is essential for lens development in mice and acts upstream of the transcription factor and early lens placode marker *Sox2 *[29,30], which in turn directly regulates crystallins [31]. Finally, BMP7 is also expressed in the developing lens and regulates expression of *Pax6*, which also regulates crystallins [29,30].

We have previously used the transgenic line N1, in which noggin overexpression is controlled by the inducible *Hsp70 *heat shock promoter, to demonstrate a requirement for functional BMP signalling in limb and tail regeneration in *Xenopus laevis *[17,20]. Both tail and limb regeneration involve epimorphic type regeneration, the regrowth of a patterned organ comprised of multiple tissues. Here, we use the same line to show that a distinct type of regenerative process, that of transdifferentiation of cornea to lens, also depends on active BMP signalling. Noggin overexpression, which blocks BMP signalling, appeared to have no effect on the early corneal thickening and cell shape changes associated with wound healing. However, prolonged inhibition of BMP signalling prevented the subsequent progression of cells to transdifferentiating aggregates that eventually form the new lens. Instead, the extra cells seem to become hypertrophic and die. Cellular hypertrophy was also observed in the AEC of poorly regenerating *Xenopus *limb buds [32], suggesting that BMP may be a survival signal for cells during regeneration.

In support of this, we have shown that increased expression of *Nipsnap1*, a known direct BMP target gene [33], is associated with CLT. The expression of *Nipsnap1 *in developing lens suggests that it may act downstream of BMPs in specifying the lens fate. In another study of *Xenopus *CLT, Malloch et al detected transcripts from a gene annotated as similar to BMP5 [15]. CLT in *Xenopus *differs from the better studied Wolffian regeneration of the lens in newts, which takes place via dedifferentiation of the pigmented epithelial cells (PECs) of the dorsal iris rather than transdifferentiation from the more closely related cornea [2]. Despite this difference, similar signals may be involved in the process. In a recent study, a large number of ESTs were generated from *Cynops pyrrhogaster *dorsal iris PECs undergoing dedifferentiation after lentectomy [34]. In this dataset, crystallins were not identified, suggesting that the dedifferentiated PECs had not yet begun to transdifferentiate into lens cells. Maki and colleagues detected multiple members of the BMP and TGFβ growth factor pathways but report that components of the Wnt, Fgf and hedgehog signalling pathways were not detected. In newts, however, BMP inhibition leads to enhanced regeneration, in conflict with our observations in *Xenopus*. Functional evidence showed that inhibition of BMP induces formation of a lens from the ventral iris, which does not normally regenerate [35]. This difference in the role of BMP may reflect inherent differences between Wolffian regeneration in newts and CLT in *Xenopus*, possibly due to the requirement for dedifferentiation in the newt.

### Wnt signalling pathway components are upregulated during lens regeneration

Wnt/β-catenin signalling (via the canonical pathway) is known to be important in driving lens cell differentiation in mammals, with several Wnt ligands, along with their receptors (Frizzled family) and the Lrp5/6 co-receptors expressed during development of the lens [36-38]. Reporter strains in mice have shown a period of Tcf/Lef activity in the lens epithelium as it develops [39,40], and it is thought that canonical Wnt signalling is required for initial formation of the lens epithelium. In contrast, the Wnt/PCP pathway is thought to play a role in the later lens fibre differentiation from the lens epithelium [41]. Regulation of Wnt signalling via inhibition by secreted frizzled related proteins (Sfrp) also occurs in mammalian lens development [38]. *Sfrp2 *expression under a lens specific crystallin promotor in mice led to cataract formation [42].

Here, we present evidence that the developmental role of canonical wnt signalling in mammalian anterior eye formation is recapitulated in lens regeneration from the cornea in *Xenopus*. Gene ontology analysis of our RAG list demonstrated that regeneration of the lens in *Xenopus *is accompanied by significantly increased expression of several components of the Wnt signalling pathway. Although the gene ontology for the CRY list suggested involvement of Wnt/PCP pathway, this result is entirely due to the expression of a single Wnt receptor, Frz7, and there is therefore insufficient evidence to implicate Wnt/PCP rather than canonical Wnt signalling, in CLT. The antagonist Sfrp2 was also recovered from the CRY list and was expressed at 1.5 fold higher levels in regenerating CLT corneas than in sham operated controls.

Wnt signalling components were also recovered from an EST collection generated from tissue undergoing the early stages of CLT. *Wnt7b *and two genes related to the Wnt modulator *Sfrp5 *were identified by Malloch and colleagues [15]. Furthermore, Wolffian regeneration of the newt lens, while occurring from the neural crest derived pigmented epithelial cells of the dorsal iris rather than the cornea, may well employ some of the same mechanisms (Reviewed in [43]). Experiments have demonstrated that if Wnt is made available, the ventral iris can also regenerate a lens [44].

### *Pitx *genes, which may be Wnt targets, are upregulated during lens regeneration

Three members of the bicoid related homeobox transcription factor Pitx were captured in the RAG list: Pitx2, Pitx2a and Pitx1. A fourth, Pitx3, was captured in the CRY list and was expressed in lens tissue as well as 1.4 × higher in corneas undergoing CLT than in control corneas. Pitx factors have not previously been implicated in vertebrate regeneration although a recent report identified a role in asexual reproduction/regeneration of the ascidian *Botryllus schlosseri *[45]. Mutations in Pitx genes in humans are known to cause eye developmental defects, particularly affecting the anterior eye structures: cornea, iris and lens. *Pitx2 *mutations cause Axenfeld-Rieger syndrome type 1, a congenital malformation syndrome affecting the anterior eye [46,47] iridogoniodysgenesis type 2 (iris hypoplasia) [48], Peters' anomaly (defective cornea) [49] and ring dermoid of the cornea [50]. Mutations in the related gene *Pitx3 *are known to cause congenital cataracts [51]. *Pitx2 *is induced by the canonical Wnt signalling pathway directly via Lef1 [52]. Pitx genes may therefore act downstream of Wnt signalling in lens regeneration in *Xenopus*.

### Lens crystallins are markers of differentiation and are expressed during CLT

Eleven probesets representing seven UniGenes belonging to the lens crystallin family were used to search for genes with similar patterns of expression across the nine datasets. Searching our microarray data for similarly expressed genes uncovered three more crystallins: *Cryaa, cryba2 *and *crybb3 (similar)*. Of these, only *cryba2 *showed a significantly higher expression in corneas undergoing CLT than in sham-operated corneas, i.e. a regenerative response. The expression of three selected crystallin genes was observed at different times during the process of embryonic lens formation (Figure 4). The α-crystallin *cryaa *was expressed late, between lens stage 4 and 5 according to McDevitt and Brahma [53]. The β-crystallin *Cryba1 *was the earliest of the three to be expressed in the lens placode, beginning at lens stage 1-2 and the γ-crystallin *crygb *was expressed slightly later at lens stage 3-4. This pattern of developmental expression is somewhat reflected in the corneas undergoing CLT, with α-crystallins unchanged between regenerating and sham operated corneas and all identified β and γ-crystallins on the array being upregulated during CLT. Therefore we believe we have captured corneas in the act of transdifferentiating, just as the first crystallins become expressed. Furthermore, we observe a correlation in timing of α, β and γ-crystallin expression that reflects that seen during lens development. However, others have reported that the timing of crystallin expression differs between regeneration and development [54].

### Genes associated with congenital cataract formation have similar expression profiles to crystallins in our microarray data

Formation of congenital cataract often results from mutation of genes involved in the formation of the anterior eye, which includes the lens. Many cases of congenital cataract result from mutations in the crystallin genes discussed above. Our search for regeneration specific gene upregulation revealed changes in several other genes known to be involved the formation of congenital cataracts. One such gene, *Pitx3*, is discussed earlier. The b-Zip transcription factor L-maf ranks 31^st ^in the CRY list, and is 2-fold upregulated in corneas undergoing CLT relative to sham operated controls (Figure 3). *L-maf *is expressed in the lens placode and later in fibre cells in *Xenopus*, and directly activates the expression of several lens crystallins [55]. Mutations in a human homologue of this gene cause a type of congenital cataract called CCA4 (OMIM#610202) [56].

Lens intrinsic membrane protein 2 (Lim2) is ranked 17^th ^on the CRY list and a potential homologue, Lim2 similar, is ranked 13^th^. *Lim2*, but not its homolgue, was upregulated in CLT. Lim2 protein is very abundant in the human lens, and mutations in Lim2, also known as MP19, are associated with congenital or early onset cataract [23]. The forkhead transcription factor Lens1 (FoxE3) was ranked 218th in the CRY list and expression was 1.6 fold higher in CLT corneas than in sham operated controls. FoxE3 is associated with ocular dysgenesis and cataracts in humans (OMIM#601094)[57].

Cugbp1 is an RNA binding protein targeting CUG repeats. *Xenopus laevis *appears to have two homolgues of this gene, *Cugbp1b *was ranked 8^th ^in the CRY list and expression was 2.4 fold higher in CLT corneas relative to controls, and *Cugbp1a *was ranked 14^th ^with a 1.5 fold higher relative expression. While Cugbp1 is not directly linked to the formation of cataracts, expansion of CTG repeats in the 3'UTR of the human *DMPK *gene cause myotonic dystrophy, a form of adult muscular dystrophy that is accompanied by cataract formation [58]. Here, we have shown that *Cugbp1b *is expressed specifically in the forming lens and is upregulated during CLT, suggesting it could be involved in the pathology of myotonic dystrophy. However, the *Cugbp1 *duplication appears to be unique to *Xenopus laevis *and an eye developmental role has not yet been described for these CUG binding proteins in other vertebrates.

### Genes associated with pluripotency are not upregulated in three day CLT tissue but chromatin modification may occur

Recent transcriptome analysis of Wolffian regeneration of the newt lens identified several genes associated with reprogramming, such as histone deacetylases and the oncogene c-myc, highly suggestive of dedifferentiation [34,59]. Furthermore, expression of a subset of pluripotency associated genes (*Klf4, Sox2 *and *c-myc*) was found to be increased during newt lens and limb regeneration [60]. In contrast, recent evidence has shown that pluripotency genes were not up regulated during zebrafish fin regeneration, although reduction of either Oct4 or Sox2 activity prevented fins from regenerating [61]. Our microarray data, while limited to only one stage of the process, suggests that CLT occurs *without *dedifferentiation in *Xenopus*. Thirteen genes associated with pluripotency were present on the GeneChip array. However, with the exception of *Sox2*, none of these were up regulated during CLT (Figure 7). *Sox2 *was expressed at highest levels in the lens samples but was not significantly elevated in corneas undergoing CLT compared to sham operated corneas. *Sox2 *then may be involved in lens differentiation but does not seem to be indicative of dedifferentiation in this case. Similarly, *Sox2 *expression was found in limbs and tails before amputation [61]. Another pluripotency associated gene, *Fut6*, was significantly upregulated in sham operated corneas when compared to corneas undergoing CLT (3.7x), with no expression in lens. Christen *et al *recently showed the same result in *Xenopus *limb blastema vs. pseudoblastema, with what they call *Fut1 *(the same gene) being upregulated regardless of regenerative success [61]. While limited to a single timepoint in the regenerative process, our results show no evidence for pluripotency gene up regulation during CLT and we therefore suggest that returning to a pluripotent state is not part of the CLT process, unlike the lens regeneration from newt PECs. We do note, however, that genes associated with chromatin assembly and disassembly were statistically overrepresented in the RAG list, suggesting that epigenetic changes may be taking place during CLT.

## Conclusions

We have shown a functional role for BMP signalling in the process of lens regeneration from the cornea (CLT) in *Xenopus laevis *tapoles, and identified a new role for *Nipsnap1*, a of BMP signalling, in the process of lens development. Furthermore, we present strong evidence for the involvement of Wnt signalling and Pitx transcription factors in CLT. Our microarray analysis has identified many genes that are involved in lens pathology, in particular the development of congenital cataract. Finally, we have shown that although there may be alterations to chromatin, there is no evidence for a return to pluripotency, or dedifferentiation, seen in 3 day corneas undergoing CLT.

## Methods

### Transgenic frogs

The *N1 *stable line of transgenic *Xenopus *has been previously described [17]. Briefly, the animals contain a transgene (*Hsp70:Noggin*; *γ-Crystallin:GFP) *comprised of two linked parts, the first containing *X. laevis *Noggin coding sequence under the control of the *Hsp70 *promoter, and the second the green fluorescent protein (GFP) coding sequence under the control of the lens specific promoter *γ-crystallin*. The line is derived from a single insertion founder made by sperm nuclear injection using the method of [62] Kroll and Amaya 1996 modified as in Beck et al (2003) [17]. All animal experiments were subject to New Zealand's animal welfare standards for vertebrates and were reviewed by the University of Otago Animal Ethics Committee (AEC). The AEC approved all experiments under protocols AEC78/06 and AEC78/09.

### Lentectomy

Stage 50-51 tadpoles were anaesthetised in MS222 (1/4000 w/v) in 0.1 × MMR, then placed on their left sides on damp paper towels for surgery. The outer cornea was first snipped with Vannas iridectomy scissors from the posterior side and cut dorsally and ventrally before lifting up as a flap. The inner cornea was ruptured and the lens gently removed using forceps, and the flap of cornea and epidermis was replaced gently over the eye. Animals were allowed to recover in 0.1 × MMR overnight before returning to a marine biotech aquarium and fed as normal. For sham-operated animals, the flap of outer cornea/epidermis was raised as for lentectomy but then replaced without further intervention.

### Histology

Tadpoles were fixed overnight in cold ethanol/glycine fixative (70% ethanol, 15 mM glycine pH 2.0) at -20°C, dehydrated in methanol and embedded in paraffin wax. 5 μm sections were cut using a Leica microtome and stained with haematoxylin and eosin. At least five animals were sectioned for each timepoint and condition reported.

### Microarray analysis

St. 50-51 tadpoles were either subjected to lentectomy or sham operated as described above. After three days, corneas (containing some epidermis) from lentectomised eyes (R) or sham operated eyes (S) were dissected from tadpoles under anaesthesia in MS222 using Vannas iridectomy scissors and fine forceps and transferred to small pieces of dry Whatman 3 M filter paper, cut with a hole punch, in groups of 8-10. The paper discs were immediately transferred to RNA later in a 1.5 ml centrifuge tube and stored on ice until RNA isolation. Groups of 8-10 dissected lenses (L) were placed directly in RNA*later *(Qiagen). Three biological replicate samples were prepared in each case and RNA was isolated using an RNaqueous micro kit (Ambion) with brief manual homogenisation in buffer before removing the paper discs manually. An amplification step (*in vitro *transcription) was performed using 50 ng of starting material and the samples were labelled with Biotin using a Nugen Ovation kit according to the manufacturers instructions. Three biological replicate pools for each treatment (L, R and S) were hybridised to *Xenopus laevis *GeneChips (Affymetrix, version 1) and washed using protocol WS2v4_450 before scanning on a 7G Plus GeneChip Scanner 3000 (Affymetrix). Data was normalised for the nine samples using an RMA algorithm with the software GenePattern. Heatmaps were prepared in Microsoft Excel using a custom macro to colour cells according to their values.

The data discussed in this publication have been deposited in NCBI's Gene Expression Omnibus (GEO) [63] and are accessible through GEO Series accession number GSE28014.

### Annotation and gene naming

Annotation was done manually by searching NCBI UniGene with the accession number of the source sequence used to design the Affymetrix probesets. Gene nomenclature follows that used by UniGene: where this differs from common usage, it has been highlighted in the text where practical. Genes that could not be annotated confidently are marked as *transcribed*. Genes with high similarity to a known gene are marked (similar) or (sim).

### Gene ontology analysis

*Xenopus *TC (Tentative Consensus) numbers and Gene Ontology (GO) assignments for biological function were obtained for the Affymetrix *Xenopus laevis *GeneChip using Resourcerer v13.0 [64], December 2006 release. Lists of TC numbers were generated for both CRY and RAG lists and duplicate TC numbers (arising when the GeneChip contained multiple probe sets for one gene) removed using the online BAR duplicate remover tool [65]. Genemerge v1.2 [66] was used to determine GO groups that were statistically over-represented in either list compared to the genes represented on the array.

### Q-rtPCR

Primers for quantitative real time PCR were designed, where possible, to different exons, to avoid amplification of genomic DNA. The NCBI program Spidey [67] was used to predict intron-exon boundaries by comparing *X. laevis *cDNA sequence to *X. tropicalis *genomic and transcript sequence from the Joint Genome Institute [68]. Primer sequences, annealing temperatures and product sizes can be found in additional file 3.

### Cloning and ISH

Primer pairs for amplification of cDNA can be found in additional file 3.

Crystallins were amplified from reverse transcribed st 50 lens RNA and all other cDNAs from reverse transcribed RNA isolated from mixed embryo stages (13, 19, 33 and 37), using Mango TAQ (Bioline). PCR products were cloned directly into T-tailed PCRII*script *vector using a TOPO kit (Invitrogen) and transformed into chemically competent TOP10 E.coli (Invitrogen). Insertions were verified by DNA sequencing, performed by Otago University's Gene Analysis Service. Digoxygenin labelled ribonucleotide probes were made by linearising plasmids with *XhoI *and transcribing using SP6 polymerase labelled with digoxigenin-UTP labelling mix (Roche). DNase I (Invitrogen) was used to remove templates following transcription and the probes were precipitated with 2.5 M LiCl. Whole-mount in situ hybridisation of albino embryos and tadpoles was performed as described previously [69]. Proteinase K treatment was 10 μg/ml for 10 minutes for embryos.

## Authors' contributions

RCD performed microarray hybridisation, analysis and validation. CWB designed the study, performed embryo experiments and in situ hybridisation, and drafted the manuscript. Both authors read and approved the final manuscript.

## Supplementary Material

Additional file 1 **Excel spreadsheet of CRY list**. Click here for file

Additional file 2 **Excel spreadsheet of RAG list**. Click here for file

Additional file 3 **Excel spreadsheet of primers used**. Click here for file
